# Publicly available datasets on thallium (Tl) in the environment—a comment on *“Presence of thallium in the environment: sources of contaminations, distribution and monitoring methods”* by Bozena Karbowska, Environ Monit Assess (2016) 188:640 (DOI 10.1007/s10661-016-5647-y)

**DOI:** 10.1007/s10661-017-5945-z

**Published:** 2017-04-25

**Authors:** Patrice de Caritat, Clemens Reimann

**Affiliations:** 1 Geoscience Australia, GPO Box 378, Canberra, ACT 2601 Australia; 2 Research School of Earth Sciences, Australian National University, Canberra, ACT 2601 Australia; 3 Geological Survey of Norway (NGU), PO Box 6315, Sluppen, 7491 Trondheim, Norway

**Keywords:** Thallium, Geochemical survey, Environment, Sediment, Soil, Plant

## Abstract

This comment highlights a whole series of datasets on thallium concentrations in the environment that were overlooked in the recent review by Karbowska, *Environmental Monitoring and Assessment*, 188, 640, 2016 in this journal. Geochemical surveys carried out over the last few decades all over the world at various scales and using different sampling media have reported the concentration of thallium (and dozens more elements) in tens of thousands of samples. These datasets provide a ‘real-world’ foundation upon which source apportionment investigations can be based, monitoring programs devised and modelling studies designed. Furthermore, this comment also draws attention to two global geochemical mapping initiatives that should be of interest to environmental scientists.

We thank Karbowska (2016) for providing an overview of the concentration and distribution of thallium (Tl) in various environmental compartments, and attempting to synthesize the state of knowledge about biological uptake and toxicity of that element. In the abstract she states that *‘the main aim of this review was to summarize the recent data regarding the actual level of thallium content in environmental niches and to elucidate the most significant sources of thallium in the environment’*. We were, therefore, disappointed to discover that she had overlooked a number of high-quality, recent, regional-, national- and continental-scale datasets on the ‘actual’ concentration and distribution of dozens of chemical elements, including Tl, in minerogenic and organic soil horizons, sediments, water and plants, for instance. There appears to be a lack of awareness in segments of the environmental sciences and associated disciplines about these rich datasets despite their having been published in the scientific literature and government reports, publicized in newsletters, presented at numerous conferences, and, in many cases, delivered online. These datasets have by-and-large been collected by applied geochemists generally working in government geological surveys or academia over the last two decades or so. The datasets from geochemical mapping projects around the globe span nearly the full spectrum of existing conditions regarding climate, topography, ecology, morphology, geology, etc. Moreover, many of the datasets are freely available on the web. The aim of this comment is thus to raise awareness of these datasets by giving an indication of their richness and diversity, using Tl as an exemplar. These datasets illustrate the complex spatial patterns these concentrations exhibit and that ‘contamination’ is but one (generally minor) aspect of their distribution. The large-scale variations in geochemical background of any element need to be understood before additional contributing processes can be hoped to be detected and elucidated (Reimann and Caritat 2000, 2005, 2017). By better understanding the concentration ranges and the scales of heterogeneity that chemical elements, including Tl, exhibit in the near-surface inorganic and organic layers of the Earth, we hope that environmental scientists, together with geoscientists, pedologists and ecologists, will be able to develop an improved appreciation of the complexity of elemental cycles, plant and animal uptake, and toxicity of chemical elements. Based on such enhanced, ‘actual’ data-driven knowledge, better monitoring strategies and modelling designs can be developed.

Table 1 shows a statistical summary of some representative geochemical datasets available on Tl concentrations that were overlooked by Karbowska (2016). The table details the region surveyed, sampling medium, basic analysis details including the lower limit of detection (LLD), as well as the minimum, median, and maximum concentrations reported. It is not the purpose here to give a complete overview of the methods, results and interpretations of these datasets, many of which have been published elsewhere and more undoubtedly are yet to come. We invite the reader to refer to the cited primary source, and references therein, to obtain all the available detail about sample media, sampling strategy, sample preparation, analysis methods, etc. We limit our scope here to solid terrestrial materials from rocks to soils to plants, and aqueous media such as stream water and groundwater, for the sake of brevity. Although the table summarises data from over 120,000 samples, it is by no means exhaustive, and represents just a sample of what data could be quickly garnered from a brief search. It is clear from Table 1 that Tl concentration in terrestrial environments spans a large range, more than three orders of magnitude for the median values; for any given medium within a surveyed region, a similar range commonly is observed. It is therefore misleading to use a single value of Tl, say in soil, to represent a starting point for toxicological studies/models.

**Table 1:** Summary data from selected geochemical surveys with published Tl data. Projects are grouped by main sampling media. See footnote for sources

Project	Country/region	Ref	Area covered	*N*	Sampling medium	Depth	Fraction	Digestion	Analysis	LLD	Min	Median	Max
Rock, soil and sediment (concentrations in mg/kg)													
NASGL	USA	1	7.8 × 10^6^ km^2^	4857	Topsoil	A horizon	<2 mm	HCl-HNO3-HClO4-HF	ICP-MS	0.1	<0.1	0.4	11.5
				4841	Topsoil	0–5 cm	<2 mm	HCl-HNO3-HClO4-HF	ICP-MS	0.1	<0.1	0.4	8.8
NGSA	Australia	2	6.2 × 10^6^ km^2^	1190	Catchment outlet sediment	0–10 cm	<2 mm	Aqua regia	ICP-MS	0.02	<0.02	0.08	0.49
				1179	Catchment outlet sediment	“	<75 μm	Aqua regia	ICP-MS	0.02	<0.02	0.12	0.46
				1191	Catchment outlet sediment	~60–80 cm	<2 mm	Aqua regia	ICP-MS	0.02	<0.02	0.1	0.43
				1182	Catchment outlet sediment	“	<75 μm	Aqua regia	ICP-MS	0.02	<0.02	0.14	0.57
				1191	Catchment outlet sediment	0–10 cm	<2 mm	MMI	ICP-MS	0.0005	<0.0005	<0.0005	0.0191
GEMAS	Europe	3	5.6 × 10^6^ km^2^	2108	Agricultural land soil	0–20 cm	<2 mm	Aqua regia	ICP-MS	0.005	<0.005	0.12	2.45
				2023	Grazing land soil	0–10 cm	<2 mm	Aqua regia	ICP-MS	0.005	<0.005	0.11	2.46
				2108	Agricultural land soil	0–20 cm	<2 mm	MMI	ICP-MS	0.0005	<0.0005	0.0006	0.017
FOREGS	Europe	4	4.2 × 10^6^ km^2^	840	Topsoil	0–25 cm	<2 mm	HCl-HNO3-HClO4-HF	ICP-MS	0.01	0.05	0.66	24.0
				783	Subsoil	>50 cm	<2 mm	HCl-HNO3-HClO4-HF	ICP-MS	0.01	0.01	0.67	21.3
				797	Stream sediment	NA	<150 μm	HCl-HNO3-HClO4-HF	ICP-MS	0.02	<0.02	0.37	7.9
				743	Floodplain sediment	0–25 cm	<2 mm	HCl-HNO3-HClO4-HF	ICP-MS	0.02	<0.02	0.37	3.5
China	China	5	9.6 × 10^6^ km^2^	862	Topsoil	0–20 cm	NR	NR	ICP-MS	0.02	0.036	0.58	2.38
S China	S China	6	2.3 × 10^6^ km^2^	5244	Stream sediment	NA	<0.22 mm	HCl-HNO3-HClO4-HF	ICP-MS	0.003	0.039	0.64	2.96
BSS	N Europe	7	1.8 × 10^6^ km^2^	747	Agricultural land soil—top	0–25 cm	<2 mm	HCl-HNO3-HClO4-HF	ICP-MS	0.1	<0.1	0.38	2.5
				747	Agricultural land soil—bottom	~50–75 cm	<2 mm	HCl-HNO3-HClO4-HF	ICP-MS	0.1	<0.1	0.39	2.7
Barents	NW Europe	8	1.6 × 10^6^ km^2^	1357	Organic soil (O horizon)	~0–3 cm	<2 mm	Conc. HNO3	ICP-MS	0.01	0.01	0.11	0.75
				1342	Mineral soil (C horizon)	>50 cm	<2 mm	Aqua regia	ICP-AES	0.01	<0.01	0.32	9.79
Spain	Spain	9	505 × 10^3^ km^2^	13,987	Stream sediment	0–10 cm	<150 μm	HCl-HNO3-HClO4-HF	ICP-MS	0.05	<0.05	0.48	33.9
				12,325	Stream sediment	0–10 cm	<150 μm	Aqua regia	ICP-MS	0.02	<0.02	0.10	12.4
				13,505	Topsoil	0–20 cm	<70 μm	HCl-HNO3-HClO4-HF	ICP-MS	0.05	<0.05	0.62	28.1
				13,505	Topsoil	0–20 cm	<70 μm	Aqua regia	ICP-MS	0.02	<0.02	0.15	16.1
				7682	Subsoil (C horizon)	20–40 cm	<70 μm	HCl-HNO3-HClO4-HF	ICP-MS	0.05	<0.05	0.64	20.2
				7682	Subsoil (C horizon)	20–40 cm	<70 μm	Aqua regia	ICP-MS	0.02	<0.02	0.18	16.2
Sweden	Sweden	10	450 × 10^3^ km^2^	2578	Till (mineral soil, C horizon)	C horizon	<63 μm	Aqua regia	ICP-MS	0.1	<0.1	0.16	1.8
Kola	NW Europe	11	188 × 10^3^ km^2^	617	Organic soil (O horizon)	0–5 cm	<2 mm	Conc. HNO3	ICP-MS	0.01	0.02	0.092	0.56
Czech Republic	Czech Republic	12	79 × 10^6^ km^2^	259	O horizon	O horizon	<2 mm	Conc. HNO3	ICP-MS	0.01	0.12	0.31	1.3
N-Trøndelag	Norway	13	25 × 10^3^ km^2^	752	Organic soil (O horizon)	O horizon	<2 mm	Aqua regia	ICP-MS	0.01	0.019	0.08	0.55
				752	Mineral soil (C horizon)	C horizon	<2 mm	Aqua regia	ICP-MS	0.02	<0.02	0.07	1.3
NGU/USGS	S Norway	14	200 km transect	44	Organic soil (O horizon)	O horizon	<2 mm	Aqua regia	ICP-MS	0.02	0.04	0.16	0.57
				44	Mineral soil	C horizon	<2 mm	Aqua regia	ICP-MS	0.02	0.02	0.09	0.35
GEOS	Norway (Oslo)	15	120 km transect	43	Bedrock	Outcrop	WR	Aqua regia	ICP-MS	0.02	<0.02	0.06	3.4
				40	Organic soil (O horizon)	O horizon	<2 mm	Aqua regia	ICP-MS	0.02	0.1	0.2	0.6
				40	Mineral soil (B horizon)	B horizon	<2 mm	Aqua regia	ICP-MS	0.02	0.02	0.12	1.5
				40	Mineral soil (C horizon)	C horizon	<2 mm	Aqua regia	ICP-MS	0.02	0.02	0.12	1.4
Barents Pilot	NW Europe	16	9 catchments over 1.5 × 10^6^ km^2^	97	Organic soil (O horizon)	O horizon	<2 mm	Conc. HNO3	ICP-MS	0.01	0.03	0.12	0.64
				97	Organic soil (O horizon)	O horizon	<2 mm	Ammonium acetate	ICP-MS	0.03	<0.03	0.03	0.4
				97	Mineral soil (C horizon)	C horizon	<2 mm	HCl-HNO3-HClO4-HF	ICP-MS	0.03	0.05	0.29	0.77
Urban soil (concentrations in mg/kg)													
Tampere	Finland	17	~164 km2	359	Topsoil	0–10 cm	<2 mm	Aqua regia	ICP-MS	0.03	0.09	0.3	0.89
Hamar	Norway	18	~65 km2	369	Topsoil	0–5 cm	<2 mm	Aqua regia	ICP-MS	0.02	<0.02	0.1	1.1
Trondheim	Norway	19	~84 km2	327	Topsoil	0–2 cm	<2 mm	Aqua regia	ICP-MS	0.1	<0.1	0.1	0.6
Karlstad	Sweden	20	~29 km2	306	Topsoil	0–10 cm	<2 mm	Aqua regia	ICP-MS	0.02	0.02	0.08	3.64
Stassfurt	Germany	21	~21 km2	479	Topsoil	0–20 cm	<2 mm	Total	AAS	0.1	<0.1	0.57	4.34
Sisak	Croatia	22	~65 km2	144	Topsoil	0–10 cm	<2 mm	Aqua regia	ICP-MS	0.02	0.05	0.15	0.62
Idrija	Slovenia	23	~3 km2	45	Topsoil	0–10 cm	<2 mm	Aqua regia	ICP-MS	0.02	0.09	0.3	0.63
				45	Subsoil	10–20 cm	<2 mm	Aqua regia	ICP-MS	0.02	0.08	0.33	0.63
Vegetation (concentrations in mg/kg)													
Barents	NW Russia + Finland	8	1.6 × 10^6^ km^2^	1346	Moss (*Hylocomium spl*.)	NA	NA	Conc. HNO3	ICP-MS	0.005	<0.005	0.03	0.38
Kola	NW Europe	11	188 × 10^3^ km^2^	598	Moss	NA	NA	Conc. HNO3	ICP-MS	0.004	<0.004	0.023	0.35
Germany	West Germany	24	~249 × 10^3^ km^2^	1006	Moss	NA	NA	Conc. HNO3	ICP-MS	0.001	<0.001	0.039	0.69
Czech Republic	Czech Republic	25	79 × 10^3^ km^2^	280	Moss	NA	NA	Conc. HNO3	ICP-MS	0.005	0.01	0.04	0.5
				265	Grass	NA	NA	Conc. HNO3	ICP-MS	0.0005	0.0009	0.005	0.42
				254	Spruce needles, 1st year	NA	NA	Conc. HNO3	ICP-MS	0.0005	0.0008	0.011	0.31
				254	Spruce needles, 2nd year	NA	NA	Conc. HNO3	ICP-MS	0.0005	0.0016	0.035	0.28
NGU/USGS	S Norway	14	Transect 200 km	46	Heather	NA	NA	Aqua regia	ICP-MS	0.02	0.3	0.8	2.2
				46	Juniper	NA	NA	Aqua regia	ICP-MS	0.02	<0.02	<0.02	0.04
				45	Birch leaves	NA	NA	Aqua regia	ICP-MS	0.02	<0.02	<0.02	0.15
				45	Willow leaves	NA	NA	Aqua regia	ICP-MS	0.02	<0.02	<0.02	0.22
Barents pilot	NW Europe	16	9 catchments over 1.5 × 10^6^ km^2^	70	Moss (*Hylocomium spl*.)	NA	NA	Conc. HNO3	ICP-MS	0.005	0.01	0.05	0.21
				70	Moss (*Pleurozium schr.*)	NA	NA	Conc. HNO3	ICP-MS	0.005	0.007	0.04	0.16
				51	Blueberry leaves	NA	NA	Conc. HNO3	ICP-MS	0.005	<0.005	<0.005	0.007
				67	Cowberry leaves	NA	NA	Conc. HNO3	ICP-MS	0.005	<0.005	<0.005	0.05
				47	Crowberry	NA	NA	Conc. HNO3	ICP-MS	0.005	<0.005	<0.005	0.006
				53	Birch leaves	NA	NA	Conc. HNO3	ICP-MS	0.005	<0.005	<0.005	0.03
				23	Willow leaves	NA	NA	Conc. HNO3	ICP-MS	0.005	<0.005	<0.005	<0.005
				38	Pine needles	NA	NA	Conc. HNO3	ICP-MS	0.005	<0.005	0.03	0.11
				42	Spruce needles	NA	NA	Conc. HNO3	ICP-MS	0.005	<0.005	0.024	0.26
Water (concentrations in μg/L)													
EGG	Europe, including Russia	26	Scattered over 10 × 10^6^ km^2^	884	Deep groundwater (bottled mineral water)	NA	Unfiltered	Conc. HNO3	ICP-MS	0.002	<0.002	0.004	2.2
EGG	Europe	26	Scattered over 5 × 10^6^ km^2^	579	Tap water	NA	Unfiltered	Conc. HNO3	ICP-MS	0.002	<0.002	0.004	1.1
FOREGS	Europe	6	4.2 × 10^6^ km^2^	807	Stream water	NA	<0.45 μm	Conc. HNO3	ICP-MS	0.002	<0.002	0.005	0.22
Barents	NW Europe	8	1.6 × 10^6^ km^2^	1365	Stream water	NA	<0.45 μm	Conc. HNO3	ICP-MS	0.001	<0.001	0.003	0.23
Norwegian groundwater	S-Norway	27	~200 × 10^3^ km^2^	476	Hardrock groundwater	NA	<0.45 μm	Conc. HNO3	ICP-MS	0.002	<0.002	0.007	0.25
Oppdal	Norway	28	2 × 10^3^ km^2^	200	Stream water	NA	<0.45 μm	Conc. HNO3	ICP-MS	0.001	0.0024	0.012	0.03

*AAS* atomic adsorption spectrometry, *Conc.* concentrated, *ICP-AES* inductively coupled plasma-atomic emission spectrometry; *ICP-MS* inductively coupled plasma-mass spectrometry, *LLD* lower limit of detection, *MMI* mobile metal ion®, *NA* not applicable, *NR* not reported, *WR* whole rock

Footnote: sources

1 North American Soil Geochemical Landscapes (Smith et al. 2014)

2 National Geochemical Survey of Australia (Caritat and Cooper 2011)

3 Geochemical Mapping of Agricultural Soils (Reimann et al. 2014)

4 Forum of European Geological Surveys (Salminen et al. 2005)

5 Handbook of Elemental Abundance (Chi and Yan 2007)

6 Geochemical mapping of southern China (Cheng et al. 2014)

7 Baltic Soil Survey (Reimann et al. 2003)

8 Barents Geochemical Survey (Salminen et al. 2004)

9 Geochemical Atlas of Spain (Locutura et al. 2012)

10 Geochemical Atlas of Sweden (Andersson et al. 2014)

11 Kola Ecogeochemistry (Reimann et al. 1998)

12 Czech Republic humus geochemistry (Sucharova et al. 2011)

13 Nord-Trøndelag (Reimann et al. 2015a)

14 Norges Geologiske Undersøkelse/United States Geological Survey Cooperation (Reimann et al. 2015b)

15 Geology of the Oslo region (Reimann et al. 2007)

16 Barents Pilot project (Reimann et al. 2001)

17 Tampere urban geochemistry (Tarvainen et al. 2013)

18 Hamar urban geochemistry (Nygard 2014)

19 Trondheim urban geochemistry (Moe 2015)

20 Karlstad urban geochemistry (Uhlbäck et al. 2014)

21 Stassfurt urban geochemistry (Birke et al. 2011)

22 Sisak urban geochemistry (Šorša and Halamić 2014)

23 Idrija urban geochemistry (Bavec et al. 2015)

24 Moss Atlas of Germany (Siewers et al. 2000)

25 Czech Republic plant geochemistry (Suchara et al. 2011)

26 European Groundwater Geochemistry Project (Reimann and Birke 2010)

27 Norwegian groundwater (Frengstad et al. 2000)

28 Oppdal surface water (Reimann et al. 2016)

The data provided in the table highlight the substantial impact (orders of magnitude) that different digestion methods of soil samples (total vs. aqua regia vs. ammonium acetate vs. mobile metal ion), grain-size fractions, soil horizons, or even land-uses, have on the analytical results for Tl. Further, it demonstrates that there exist internally consistent datasets for quite a large number of sample media from the same survey areas, allowing the determination of which ecosystem compartments tend to be enriched in Tl, and which tend to be depleted. Some of the more successful multi-media surveys include the Kola, Barents, FOREGS (Forum of European Geological Surveys) and GEOS (Geology of the Oslo region) projects. The table shows that different plants, even when growing in the same area on the same substrate, can display substantial differences in their Tl concentrations. One extreme example is the strong enrichment (about two orders of magnitude) of Tl in heather (maximum of 2.2 mg/kg) compared to juniper (maximum of 0.04 mg/kg) detected by a NGU/USGS (Norges Geologiske Undersøkelse/United States Geological Survey) cooperation project at the southern tip of Norway (Reimann et al. 2015b).

Moreover, we can demonstrate that the reported concentrations do not vary randomly in space, but form coherent geospatial patterns that are controlled by the bedrock composition, soil forming processes (including climate and vegetation), erosion/transport/deposition at the Earth’s surface, land use (e.g. grazing), mineral deposits, and so on. As an example, Fig. 1 illustrates the distribution of Tl in surface floodplain sediments in Australia (Caritat and Cooper 2011). It is well established that Tl tends to be more abundant in felsic than in mafic rocks, e.g. average of 1.1 mg/kg in granite/granodiorite vs. 0.18 mg/kg in gabbro/basalt (Koljonen 1992). Similarly, in sedimentary rocks, clay-rich material holds more Tl than coarse-grained material, e.g. 1 mg/kg in shale/schist vs. 0.4 mg/kg in sandstone (Koljonen 1992), due to its tendency to adsorb on clay mineral surfaces. Thallium will also adsorb on iron and manganese oxy-hydroxides and organic matter (e.g. Kazantzis 2000). The most enriched common rock type is coal with an average of 3 mg/kg (Koljonen 1992). Whereas crookesite Cu_7_(Tl,Ag)Se_4_) and lorandite (TlAsS_2_) are typical but rare Tl ‘ore’ minerals, much more common minerals such as micas and K-feldspars, as well as many sulfide ores, contain traces of Tl, which is a chalcophile metal.

**Fig. 1:**
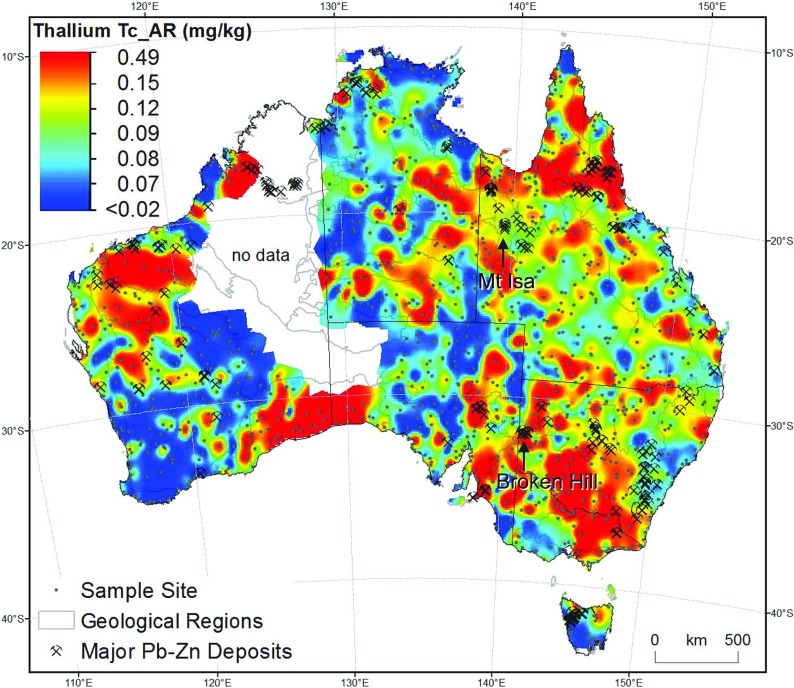
Thallium distribution (in mg/kg) in top outlet sediments (‘T’: 0–10 cm) coarse fraction (‘c’: <2 mm) after aqua regia (‘AR’) digestion over Australia (Caritat and Cooper 2011). Raster surface obtained by inverse distance weighting interpolation. Sampling sites, major Pb-Zn deposits and the geological regions of Blake and Kilgour (1998) are overlain

Thus, the distribution of Tl in surface soil is likely to reflect to a large extent the lithology and, under the right conditions, the mineralisation potential of the source/parent material. On top of that natural and spatially variable background, where heavy industry (e.g. petroleum refineries, coal-fired power plants, sulfide ore smelters, waste incinerators and cement factories; Schaub 1996; Reimann and Caritat 1998) has been present for an extended period of time, anthropogenic additions can occur. In Australia (Fig. 1), the dominant control on Tl distribution in surface sediments is geology (Reimann and Caritat 2017), particularly felsic rocks (e.g. SE Australia), iron oxide-rich bedrock (e.g. NW Australia) and clay minerals dominated sediments/weathered materials (e.g. S central Australia, interior of Australia). Some of the major base metal (e.g. Pb-Zn) sulfide ore provinces such as Broken Hill are coincident with local to regional anomalies too; however, the Mount Isa mineral province is not accompanied by a particularly remarkable Tl anomaly. The map is overwhelmingly dominated by the natural and variable background.

Figure 2 shows the regional distribution of Tl in organic soil (O horizon) of podzols in the European Arctic from the Kola Ecogeochemistry Project (Reimann et al. 1998), covering an area of 188 × 10^3^ km^2^. Here both the impact of contamination (from the Ni refinery in Monchegorsk) and ‘nature’, i.e. a strong north-to-south increasing gradient in Tl concentrations due to the changing vegetation/climate zones (from arctic tundra to boreal forest), are visible and the scale and relative importance of different processes can be judged.

**Fig. 2:**
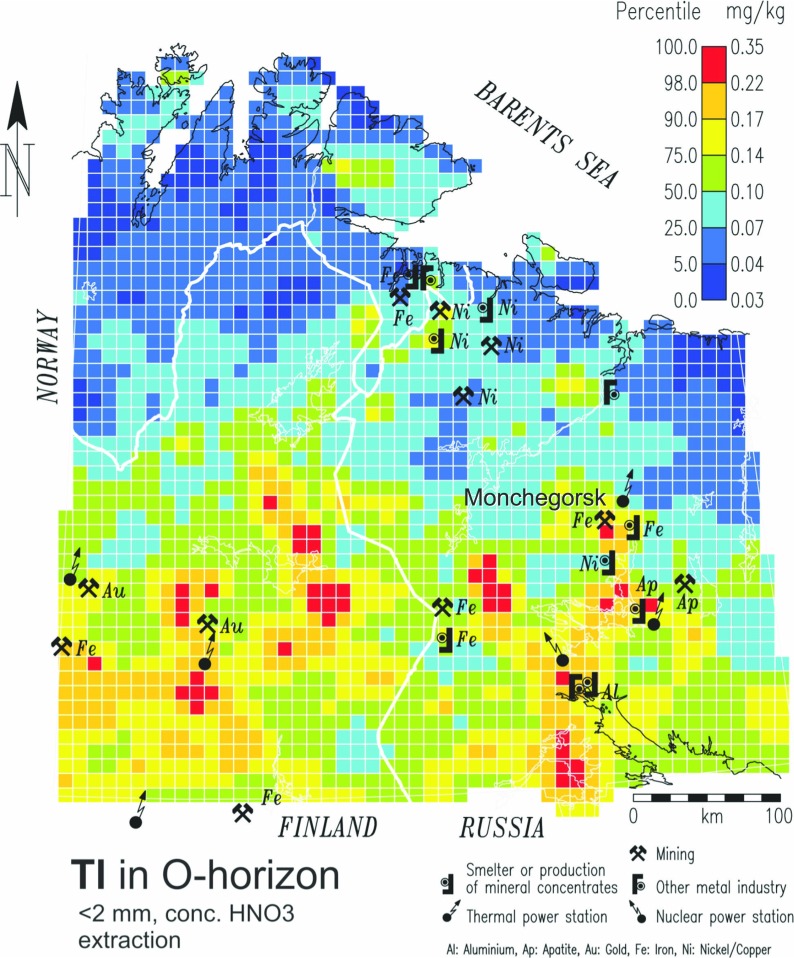
Thallium distribution (in mg/kg) in soil O horizon <2 mm fraction after concentrated HNO_3_ digestion over the Kola Ecogeochemistry study area of northern Norway, northern Finland and northwestern Russia (Reimann et al. 1998). Raster surface obtained by ordinary kriging interpolation. Major industrial sites are overlain

In Fig. 3, we show how the quantile-probability distribution of Tl in surface soil/sediment varies between two continental regions, Australia and Europe. All values <LLD have been replaced by half the LLD and form clearly visible sub-populations at the lowest concentration end. The overall Tl concentration is lower in Australia than in Europe, likely a grain-size fraction effect of the sandier material common in Australia. Note that this modest difference is only marginal in the central quantiles, say from the 20th percentile to the 85th percentile, and increases at both extremes of the distributions. It appears that there are at least two sub-populations in the Australian dataset, with a break at the ~95th percentile (~0.25 mg/kg). Above the ~99th percentile (~0.9 mg/kg), the European dataset also deviates from a relatively straight line, likely also indicating a major different sub-population. In both cases, it would be instructive to plot these sub-populations and compare them with lithology and other potential controls/sources. A final observation from Fig. 3 is that the dataset from Europe defines a much smoother distribution than that from Australia, reflecting an artefact stemming from excessive rounding of the analytical values at the lower concentration end in the latter case.

**Fig. 3:**
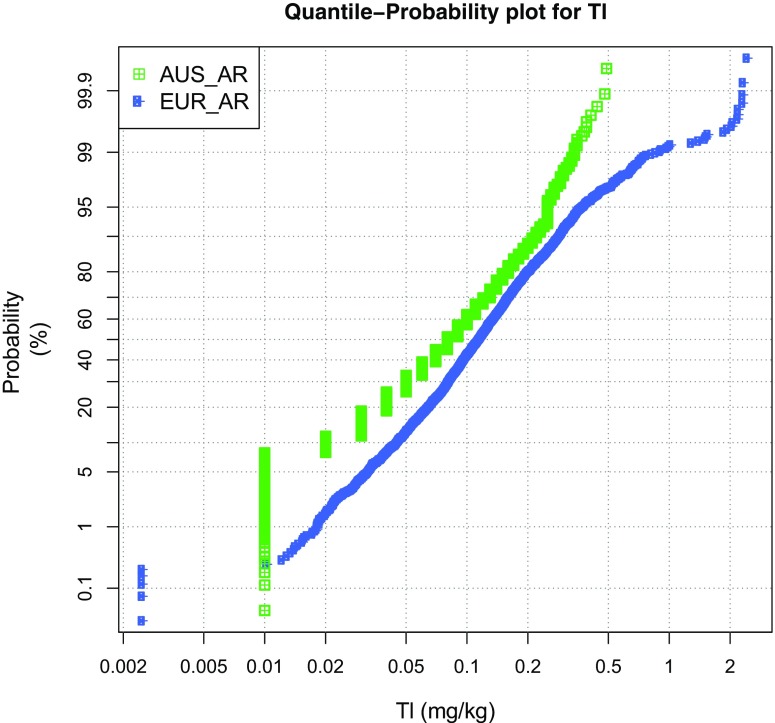
Quantile probability plot for two continental-scale geochemical datasets from Australia (Caritat and Cooper 2011) and Europe (Reimann et al. 2014)

Based on the above, we argue that it is nigh on impossible to provide a valid review of Tl, or indeed any element, in the environment, whilst ignoring such compelling datasets.

In closing, we would like to draw attention to two international initiatives concerned with geochemical mapping of continents and indeed the whole terrestrial globe. The first is the Commission for Global Geochemical Baselines established under the auspices of the International Union for Geological Sciences (IUGS). It was initially established in 1988 as an IUGS/IAGC (International Association of GeoChemistry) Task Group (Smith et al. 2012) and upgraded to Commission in 2016. Its history and, importantly, database and many more useful details can be found here: http://www.globalgeochemicalbaselines.eu/ (Accessed 29 November 2016). The second initiative is the International Center on Global-Scale Geochemistry (http://www.globalgeochemistry.com/; Accessed 29 November 2016), recently inaugurated under the auspices of UNESCO and with considerable financial support from the government of China. This Center, headquartered in Langfang, China, aims to foster knowledge and technology for the sustainable development of global natural resources and environments; to document the global concentration and distribution of chemical elements at the Earth’s surface; to educate and train the next generation of scientists; and to promote access to global-scale geochemical data. Both the Commission and the Center are working hand-in-hand to assist many more regions and countries around the planet acquiring geochemical datasets and atlases. Whilst already ~25% of the Earth’s continental surface area is covered with geochemical data at global-scale density (i.e. mainly China, Europe, the conterminous USA, and Australia), more will come into the public domain over coming years; watch this space!

## References

[CR1] Andersson M, Carlsson M, Ladenberger A, Morris G, Sadeghi M, Uhlbäck J (2014). Geochemical Atlas of Sweden.

[CR2] Bavec Š, Biester H, Gosar M, Grčman H (2015). Geochemical investigation of mercury and other elements in urban soil of Idrija (Slovenia). Journal of Geochemical Exploration.

[CR3] Birke M, Rauch U, Chmieleski J, Johnson CC, Demetriades A, Locutura J, Ottesen RT (2011). Environmental geochemical survey of the city of Stassfurt: an old mining and industrial urban area in Sachsen-Anhalt, Germany. Mapping the chemical environment of urban areas.

[CR4] Blake, D., & Kilgour, B. (1998). Geological Regions of Australia 1:5,000,000 scale [Dataset]. Geoscience Australia, Canberra. http://www.ga.gov.au/metadata-gateway/metadata/record/gcat_a05f7892-b237-7506-e044-00144fdd4fa6/Geological+Regions+of+Australia%2C+1%3A5+000+000+scale. Accessed 29 November 2016.

[CR5] Caritat, P. de, & Cooper, M. (2011). National Geochemical Survey of Australia: The Geochemical Atlas of Australia. Geoscience Australia Record 2011/20, 557 p. (2 Volumes). http://www.ga.gov.au/metadata-gateway/metadata/record/gcat_71973. Accessed 29 November 2016.

[CR6] Cheng Z, Xie X, Yao W, Feng J, Qin Z, Fang J (2014). Multi-element geochemical mapping in Southern China. Journal of Geochemical Exploration.

[CR7] Chi Q, Yan M (2007). Handbook of elemental abundance for applied geochemistry.

[CR8] Frengstad, B., Midtgård, A. K., Banks, D., Krog, J. R., & Siewers, U. (2000). The chemistry of Norwegian groundwaters: III. The distribution of trace elements in 476 crystalline bedrock groundwaters, as analyzed by ICP-MS techniques. *Science of the Total Environment, 246*, 21–40. 10.1016/s0048-9697(99)00413-1 10682374

[CR9] Karbowska B (2016). Presence of thallium in the environment: sources of contaminations, distribution and monitoring methods. Environmental Monitoring and Assessment.

[CR10] Kazantzis G (2000). Thallium in the environment and health effects. Environmental Geochemistry and Health.

[CR11] Koljonen T (1992). Geochemical Atlas of Finland, Part 2: Till.

[CR12] Locutura, J., Bel-lan, A., García-Cortés, A., & Martínez, S. (2012). *Atlas Geoquímico de España*. Instituto Geológico y Minero de España, Madrid, 592 p. ISBN: 978-84-7840-875-7.

[CR13] Moe, I. M. D. (2015). Kartlegging av forurensning i urbane løsmasser—Innhold av uorganiske miljøgifter og organiske forbindelser av polysykliske aromatiske hydrokarboner (PAH) i overflatejord fra Trondheim - utvikling og sammenligning med undersøkelsen fra 1994. MSc Thesis, NTNU (Norwegian University of Science and Technology), 155 p. + Appendices. https://brage.bibsys.no/xmlui//handle/11250/2350435. Accessed 29 November 2016

[CR14] Nygard, I. (2014). Geokjemisk kartlegging a metaller i jord i Hamar by. MSc Thesis, NTNU (Norwegian University of Science and Technology), 113 p + Appendices. https://brage.bibsys.no/xmlui/handle/11250/248014. Accessed 29 November 2016.

[CR15] Reimann C, Siewers U, Tarvainen T, Bityukova L, Eriksson J, Gilucis A, Gregorauskiene V, Lukashev VK, Matinian NN, Pasieczna A (2003). Agricultural soils in northern Europe: a geochemical atlas. Geologisches Jahrbuch, Sonderhefte, Reihe D, Heft SD 5.

[CR16] Reimann C, Birke M (2010). Geochemistry of European bottled water.

[CR17] Reimann, C., & Caritat, P. de (1998). *Chemical elements in the environment—Factsheets for the geochemist and environmental scientist*. Berlin: Springer-Verlag 398 pp.

[CR18] Reimann, C., & Caritat, P. de (2000). Intrinsic flaws of element enrichment factors (EFs) in environmental geochemistry. *Environmental Science & Technology, 34*, 5084–5091.

[CR19] Reimann, C., & Caritat, P. de (2005). Distinguishing between natural and anthropogenic sources for elements in the environment: regional geochemical surveys versus enrichment factors. *Science of the Total Environment, 337*, 91–107. 10.1016/j.scitotenv.2004.06.011 15626382

[CR20] Reimann, C., & Caritat, P. de (2017). Establishing geochemical background variation and threshold values for 59 elements in Australian surface soil. *Science of the Total Environment,* 578,633–648. 10.1016/j.scitotenv.2016.11.010 27863868

[CR21] Reimann C, Arnoldussen A, Englmaier P, Filzmoser P, Finne TE, Garrett RG, Koller F, Nordgulen Ø (2007). Element concentrations and variations along a 120-km long transect in south Norway—Anthropogenic vs. geogenic vs. biogenic element sources and cycles. Applied Geochemistry.

[CR22] Reimann, C., Äyräs, M., Chekushin, V., Bogatyrev, I., Boyd, R., Caritat, P. de, Dutter, R., Finne, T. E., Halleraker, J. H., Jaeger, O., Kashulina, G., Lehto, O., Niskavaara, H., Pavlov, V., Räisänen, M. L., Strand, T., & Volden, T. (1998). *Environmental geochemical atlas of the central Barents region*. Trondheim: Geological Survey of Norway 745 p.

[CR23] Reimann, C., Birke, M., Demetriades, A., Filzmoser, P., & O’Connor, P. (eds) (2014). Chemistry of Europe’s agricultural soils–Part A: Methodology and Interpretation of the GEMAS Data Set. Geologisches Jahrbuch, B102: 528 pp.

[CR24] Reimann, C., Birke, M., Eggen, O. A., Gasser, D., & Sandstad, S. V. (2016). Surface water geochemistry, Oppdal and Rennebu county, South Trøndelag, Norway. *Geological Survey of Norway, NGU Report, 2016.009*, 342 p

[CR25] Reimann, C., Fabian, K., Schilling, J., Roberts, D., & Englmaier, P. (2015a). A strong enrichment of potentially toxic elements (PTEs) in Nord Trøndelag (central Norway) forest soil. *Science of the Total Environment, 536*, 130–141. 10.1016/j.scitotenv.2015.07.032 26204049

[CR26] Reimann, C., Englmaier, P., Fabian, K., Gough, L., Lamothe, P., & Smith, D. (2015b). Bio-geochemical plant-soil interaction: variable element composition in leaves of four plant species collected along a south-north transect at the S-tip of Norway. *Science of the Total Environment, 506–507*, 480–495. 10.1016/j.scitotenv.2014.10.079 25437765

[CR27] Reimann, C., Koller, F., Frengstad, B., Kashulina, G., Niskavaara, H., & Englmaier, P. (2001). Comparison of the element composition in several plant species and their substrate from a 1,500,000 km2-area in Northern Europe. *Science of the Total Environment, 278*, 87–112. 10.1016/s0048-9697(00)00890-1 11669279

[CR28] Salminen, R., Batista, M. J., Bidovec, M., Demetriades, A., De Vivo, B., De Vos, W., Gilucis, A., Gregorauskiene, V., Halamic, J., Heitzmann, P., Lima, A., Jordan, G., Klaver, G., Klein, P., Lis, J., Locutura, J., Marsina, K., Mazreku, A., Mrnkova, J., O'Connor, P. J., Olsson, S. Ǻ., Ottesen, R. T., Petersell, V., Plant, J. A., Reeder, S., Salpeteur, I., Sandström, H., Siewers, U., Steenfelt, A., & Tarvainen, T. (2005). FOREGS Geochemical Atlas of Europe, Part 1—Background Information, Methodology, and Maps. Geological Survey of Finland, Espoo, 690 p. http://weppi.gtk.fi/publ/foregsatlas/. Accessed 29 November 2016.

[CR29] Salminen R, Chekushin V, Tenhola M, Bogatyrev I, Glavatskikh SP, Fedotova E, Gregorauskiene V, Kashulina G, Niskavaara H, Polischuok A, Rissanen K, Selenok L, Tomilina O, Zhdanova L (2004). Geochemical atlas of the eastern Barents region.

[CR30] Schaub, G. (1996). Thallium—Environmental Health Criteria. International Programme on Chemical Safety, World Health Organization Publication. http://www.inchem.org/documents/ehc/ehc/ehc182.htm. Accessed 29 November 2016.

[CR31] Siewers, U., Herpin, U., & Strassburg, S. (2000). Schwermetalleinträge in Deutschland, Moss Monitoring 1995/96, Teil 2. Geologisches Jahrbuch, Sonderhefte heft SD 3, 103 p.

[CR32] Smith, D. B., Cannon, W. F., Woodruff, L. G., & Ellefsen, K. J. (2014). Geochemical and mineralogical maps for soils of the conterminous United States. United States Geological Survey Open-File Report, 2014–1082, 386 p.

[CR33] Smith DB, Wang X, Reeder S, Demetriades A (2012). The IUGS/IAGC task group on global geochemical baselines. Earth Science Frontiers.

[CR34] Šorša A, Halamić J (2014). Geochemical atlas of Sisak. Public library Vlado Gotovac Sisak.

[CR35] Suchara, I., Sucharova, J., Hola, M., Reimann, C., Boyd, R., Filzmoser, P., & Englmaier, P. (2011). The performance of moss, grass and 1- and 2-year old spruce needles as bioindicators of contamination: a comparative study at the scale of the Czech Republic. *Science of the Total Environment, 409*, 2281–2297. 10.1016/j.scitotenv.2011.02.003 21421258

[CR36] Sucharova J, Suchara I, Hola M, Reimann C, Boyd R, Filzmoser P, Englmaier P (2011). Linking chemical elements in forest floor humus in the Czech Republic to contamination sources. Environmental Pollution.

[CR37] Tarvainen, T., Luoma, S., & Hatakka, T. (2013). Tampereen taajama-alueen maaperän taustapitoisuudet. Geological Survey of Finland, Report, 128/2013, 29 p. http://tupa.gtk.fi/raportti/arkisto/128_2013.pdf. Accessed 29 November 2016.

[CR38] Uhlbäck, J., Andersson, M., & Ladenberger, A. (2014). Urban geokemi i Karlstad. Geological Survey of Sweden, SGU Report, 2014:25, 111 p.

